# Recombinant SFRP5 protein significantly alleviated intrahepatic inflammation of nonalcoholic steatohepatitis

**DOI:** 10.1186/s12986-017-0208-0

**Published:** 2017-08-15

**Authors:** Lili Chen, Xiaolong Zhao, Guangjun Liang, Jiuru Sun, Zhifeng Lin, Renming Hu, Peili Chen, Zhaoyun Zhang, Linuo Zhou, Yiming Li

**Affiliations:** 1 Department of Endocrinology, Huashan Hospital Fudan University, 12 Middle Wulumuqi Road, Shanghai, 200040 People’s Republic of China; 2 Shanghai Anruite Biological Medicine Technology Co., Ltd., 200 Newton Road, Zhangjiang Hi-Tech Park, Shanghai, 201210 People’s Republic of China

**Keywords:** SFRP5, Nonalcoholic steatohepatitis, Non alcoholic fatty liver disease, Chronic inflammation

## Abstract

**Background:**

Secreted frizzled-related protein 5 (SFRP5) is an anti-inflammatory adipokine modulating metabolism dysfunction. This study aims to observe the effect of recombinant SFRP5 protein on nonalcoholic steatohepatitis (NASH).

**Methods:**

We set up a prokaryotic expression system and purified the recombinant SFRP5 protein. Recombinant SFRP5 protein was further identified by SDS-PAGE, western blot, high performance liquid chromatography (HPLC), protein mass spectrometry and in vitro Wnt5a-binding test. NASH mouse model was induced by methionine and choline deficient diet (MCDD) for 2 weeks. SFRP5 treatment group received intraperitoneal injection with a dosage of 10μg/kg SFRP5 twice a day for 2 weeks. Saline was used as control. Inflammation and fatty lesion score of liver tissue pathology and serum transaminase level were compared.

**Results:**

The purity of recombinant SFRP5 protein is 90% identified by HPLC. Its molecule size is 36,096.08 tested by mass spectrometry. Recombinant SFRP5 can specifically bind with Wnt5a which verifies its activity in vitro. The endotoxin level of this recombinant protein is 0.01EU/μg-0.1EU/μg and is suitable for animal experiment. SFRP5 can significantly improve liver inflammation (SFRP5 vs. control, 1.40 ± 0.70 vs. 2.00 ± 0.47, *P* < 0.05) as well as fatty lesion scores (SFRP5 vs. control, 1.40 ± 0.97 vs. 2.20 ± 0.63, *P* < 0.05), and lower ALT and AST levels. The mRNA expression of proinflammatory adipokines (IL-1β, IL-6, TNFα and MCP-1) in liver was down-regulated significantly after SFRP5 intervention. Immunohistochemistry and quantitative PCR revealed a dramatically down-regulation of F4/80 in liver after SFRP5 treatment.

**Conclusions:**

Recombinant SFRP5 protein significantly alleviated NASH induced by MCDD.

**Electronic supplementary material:**

The online version of this article (doi:10.1186/s12986-017-0208-0) contains supplementary material, which is available to authorized users.

## Background

Due to the sedentary lifestyle and energy excess, the global pandemic of obesity further leads to an increasing prevalence of non alcoholic fatty liver disease (NAFLD) as high as approximately 30% [1, 2]. As an important component of metabolic syndrome, NAFLD is associated with cardiocerebral vascular events [2]. Moreover, NAFLD is one of the most common causes of chronic decompensated liver disease [2]. According to the natural course and pathologic features, NAFLD consists of 4 stages: simple steatosis, nonalcoholic steatohepatitis (NASH), fibrosis, and cirrhosis. NASH is the key pathophysiologic phase during NAFLD progress [3]. The hepatosteatosis complicated with lobular inflammation and fibrosis, which can further develop into cirrhosis and hepatocarcinoma [3]. In US, many of the unexplained hepatic fibrosis cases are eventually attributed to NASH [4], one third of hepatocarcinoma is associated with NASH [5]. NASH accounts for only 1.2% of liver transplantations in 2000 in the US, while the proportion rises to 7.4% till the year 2010 [6]. In China, the NASH epidemic is also associated with an increased risk for the hepatocarcinoma due to non-viral hepatits [7]. Simple steatosis is reversible. However, activation of intrahepatic inflammation and the subsequent fibrosis and cirrhosis is irreversible [3, 8]. Hence, intervention of NASH is of great importance for the prevention of NALFD-associated adverse prognosis. Currently, only vitamin E and pioglitazone have been verified to be effective for the treatment of NASH [6]. Hence, drugs with innovative mechanism and better efficacy are still to be explored.

As a novel adipokine mainly secreted from adipose tissue [9], secreted frizzled-related protein 5 (SFRP5) contains a cysteine rich domain as well as a netrin-like function domain, and plays a regulatory role in the Wnt signaling pathways. Preliminary clinical and basic research reveals that the biologic function of SFRP5 may be similar with adiponection, which exerts an anti-inflammatory role in the metabolic homeostasis [9]. For instance, serum SFRP5 level is associated with obesity and type 2 diabetes. Serum SFRP5 level of diabetic patients with obesity is lower than normal subjects, while GLP-1 agonist liraglutide intervention results in weight loss and serum SFRP5 elevation [10]. SFRP5 protein levels were significantly lower in NASH than in control subjects [11]. *SFRP5* knockout mice develop obesity and adipose inflammation [9, 12], while *SFRP5* overexpression via adenovirus could alleviate obesity, adipose inflammation and hepatosteatosis. The classical molecular mechanism of SFRP5 is to inhibit the combination of Wnt protein with its cell membrane receptors (frizzled protein) and block the downstream Wnt signaling pathways through binding with extracellular Wnt-5a [13] or Wnt-3a [14].

According to the previous research, SFRP5 plays an anti-inflammatory role in metabolic homeostasis [9, 12]. However, to date, little is known about the effect of SFRP5 on NASH. The current study is aimed to investigate the efficacy of recombinant SFRP5 for intrahepatic inflammation of NASH.

## Methods

### Cloning, expression and purification of recombinant SFRP5

The recombinant SFRP5 protein including 10 extra His amino acids at the N terminal was designed, and the whole *SFRP5* DNA sequence was synthesized. Then the *SFRP5* gene was cloned into expression plasmids to generate pET30-SFRP5 expression constructs. The nucleotide sequence of pET30-SFRP5 was determined by Sangon Biotech and found to be identical to the designed sequence.

Subsequently, SFRP5 was expressed in *E. coli* BL21DE3 cells (Novagen). The transformed bacteria were grown in LB medium at 37 °C. Growth was monitored by absorbance measurements at 600 nm (OD600). While OD600 reached 0.6, expression was induced with 1 mM IPTG. at 16 °C for 4 h. In a typical protocol cells were grown in 4 l of LB medium and the harvested cells were either stored at 37 °C or lysed on ice in 30 ml of Buffer A (25 mM Tris, pH 8.0, 100 mM NaCl, 1 mM 2-ME, 0.1% Triton X-100, 10% glycerol) containing 1 mM PMSF, EDTA-free protease inhibitor cocktail (Boehringer Mannheim), and 0.5 mg/ml lysozyme. After 3 repeats, the cells were sonicated and then centrifuged at 10,000 g for 30 min at 4 °C. The supernatant was collected. The protein purification process is hydrophobic chromatography (phenyl resin) firstly, ion exchange chromatography (DEAE resin), and affinity chromatography (Ni resin) finally. The cell lysis supernatants including 2 M (NH4)_2_SO_4_ was loaded through the phenyl resin, and the SFRP5 protein was washed by ddH_2_O. The washed SFRP5 protein was then captured by DEAE resin under cond < 5 ms/cm, pH 8.0, and eluted by 20 mM PB 50 Mm NaCl pH 8.0. Although the protein PI < 8.0, oddly it can be caputured by DEAE, non-binding with the SP resin. Finally the SFRP5 protein is bound with the Ni agarose, and washed by 5 mM imidazole. The imidazole is removed by dialysis.

### Identification of recombinant SFRP5

Purified SFRP5 was analyzed on 10% SDS–PAGE, and then underwent western blot identification. SFRP5 (SARP3) primary antibody (Santa Cruz Biotechnology, E-19, sc-14,331, goat polyclonal IgG) was employed. The concentration of primary and secondary antibodies was 1:1000. The purified SFRP5 protein was further identified through high performance liquid chromatography (HPLC) and mass spectrometry. For HPLC, the injection volume was 100ul, and the run time was 60 min. The endotoxin level of recombinant SFRP5 protein was tested by tachypleus amebocyte lysate (TAL)-LAL method [15].

### In vitro binding specificity test of recombinant SFRP5 with Wnt-5a

Purified SFRP5 protein with different concentrations were coated on 96-well plates then incubated overnight with a sufficient amount of Wnt-5a protein (WN645, R & D). Subsequently, Wnt-5a primary antibody (R & D, MAB645) was added, followed by addition of DAB-containing secondary antibody, and the OD value was measured to generate an affinity curve.

### SFRP5 intervention for methionine and choline deficient diet (MCDD) induced NASH

In this study, 30 male adult C57 mice of 4 weeks old were purchased from Shanghai Slack Laboratory Animal Co., Ltd. (Shanghai, China). The mice were fed with normal diet for 4 weeks. Then, at 8 weeks of age, mice were randomly divided into three groups: normal diet group, MCDD + saline group and MCDD + SFRP5 group. One group was fed with normal diet and the other two groups were fed with methionine and choline deficient diet (MCDD, Reasearch Diets Co., Ltd., A02082002B) for 2 weeks. In MCDD + SFRP5 group, recombinant sfrp5 protein was injected intraperitoneally 0.5μg twice daily for 2 weeks. In MCDD + saline group, saline was injected intraperitoneally twice daily as control. The mice got free access to food and water. Two weeks later, at the age of 10 weeks, the mice were sacrificed and the blood was sampled. The levels of serum ALT and AST were measured by routine biochemical method. The liver tissue was fixed with 4% neutral formaldehyde and stained with HE. In accordance with the histological scoring system for nonalcoholic fatty liver disease (Additional file 1: Table S1) [16], two experienced pathologists evaluated the liver pathology in blinded manner. All animal procedures were approved by the Animal Ethics Committee of Fudan University.

Quantitative PCR was used to detect the expression of inflammatory cytokines in liver. The hepatic Kupffer cells were stained with F4/80 antibody (1: 500) by immunohistochemistry and the expression of F4/80 mRNA in liver tissue was detected by quantitative PCR. Primer sequences for the target mouse genes are F4/80: forward primer CACTGTGACCGGGGAGAAGAAGG, reverse primer CAAGTTTGCCATCCGGTTACAGC; IL-6: forward primer GTTGCCTTCTTGGGACTGAT, reverse primer GCCATTGCACAACTCTTTTCT; TNF-α: forward primer CTGCCCCGACTACGTGCTCCTCA, reverse primer AGTTGGTCCCCCTTCTCC; MCP-1: forward primer TTCACCAGCAAGATGATCCCA, reverse primer TCCTTCTTGGGGTCAGCACA; IL-1β: forward primer GGAGAACCAAGCAACGACAAAATA, reverse primer TGGGGAACTCTGCAGACTCAAAC.

### Statistical analysis

Results are presented as mean ± SEM. Statistical analysis was performed by ANOVA and Student *t*-test (GraphPad Software, San Diego, CA, USA). A value of *P* < 0.05 was considered as statistically significant.

## Results

### Expression and purification of recombinant SFRP5

**Fig. 1:**
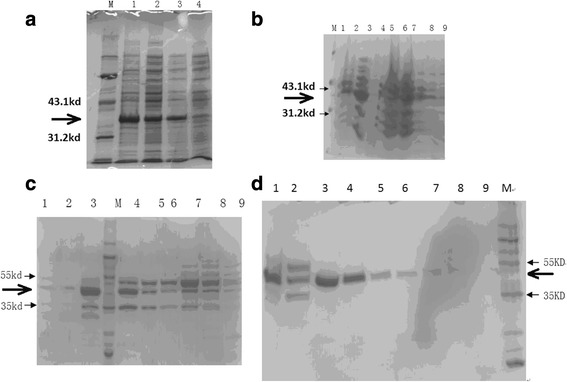
Expression and purification of SFRP5 protein. **a** Identification of pET30-SFRP5 expression. M marker (97.4kd,66.2kd,43.1kd,31.2kd,21.2kd,14.4kd). 1. Precipitate of ultrasound-disrupted *E. coli* cells induced by pET30-SFRP5 for 4 h; 2. Supernatant of ultrasound-disrupted *E. coli* cells induced by pET30-SFRP5 for 4 h; 3. Ultrasound-disrupted *E. coli* cells induced by pET30-SFRP5 for 4 h; 4. Ultrasound-disrupted *E. coli* cells. **b** The supernatant of the disrupted bacteria was subjected to hydrophobic chromatography, and the bacterial supernatant protein was added to 2 M ammonium sulfate, followed by elution. M marker (97.4kd,66.2kd,43.1kd,31.2kd,21.2kd,14.4kd). 1-9: elusion products. **c** The hydrophobic chromatography eluate was titrated to PH 8.0 and subjected to ion exchange chromatography. 1. DEAE sample before loading; 2. DEAE penetration sample; 3. 20 mM PB 50 mM NaCl PH 8.0 eluent 1; M:Marker (130kd,100kd,70kd,55kd,35kd,25kd,15kd,10kd); 4: 20 mM PB 50 mM NaCl PH 8.0 eluent 2; 5: 20 mM PB 50 mM NaCl PH 8.0 eluent 3; 6: 20 mM PB 50 mM NaCl PH 8.0 eluent 4; 7: 20 mM PB 100 mM NaCl PH 8.0 eluent 1; 8: 20 mM PB 100 mM NaCl PH 8.0 eluent 2; 9: 20 mM PB 100 mM NaCl PH 8.0 eluent 3. **d** Affinity chromatography: 20 mM PB 50 mM NaCl 8.0 pH 8.0 ion exchange chromatography eluate was subjected to nickel column affinity chromatography. 1. Ni sample before loading; 2. Ni penetration sample; 3. 20 mM PB 0.15 M NaCl 5 mM imidazole PH 8.0 eluent 1; M Marker (130kd,100kd,70kd,55kd,35kd,25kd,15kd,10kd); 4. 20 mM PB 0.15 M NaCl 5 mM imidazole PH 8.0 eluent 2; 5. 20 mM PB 0.15 M NaCl 5 mM imidazole PH 8.0 eluent 3; 6. 20 mM PB 0.15 M NaCl 30 mM imidazole PH 8.0 eluent 1; 7. 20 mM PB 0.15 M NaCl 30 mM imidazole PH 8.0 eluent 2; 8. 20 mM PB 0.15 M NaCl 30 mM imidazole PH 8.0 eluent 3; 9. 20 mM PB 0.15 M NaCl 30 mM imidazole PH 8.0 eluent 4

Recombinant SFRP5 protein was expressed using *E. coli* amplification system BL21DE3-pET30-SFRP5 (Fig. 1a). The protein supernatant of the disrupted bacteria further underwent hydrophobic chromatography (Fig. 1b), ion exchange chromatography (Fig. 1c) and affinity chromatography (Fig. 1d).

### Identification of recombinant SFRP5

**Fig. 2:**
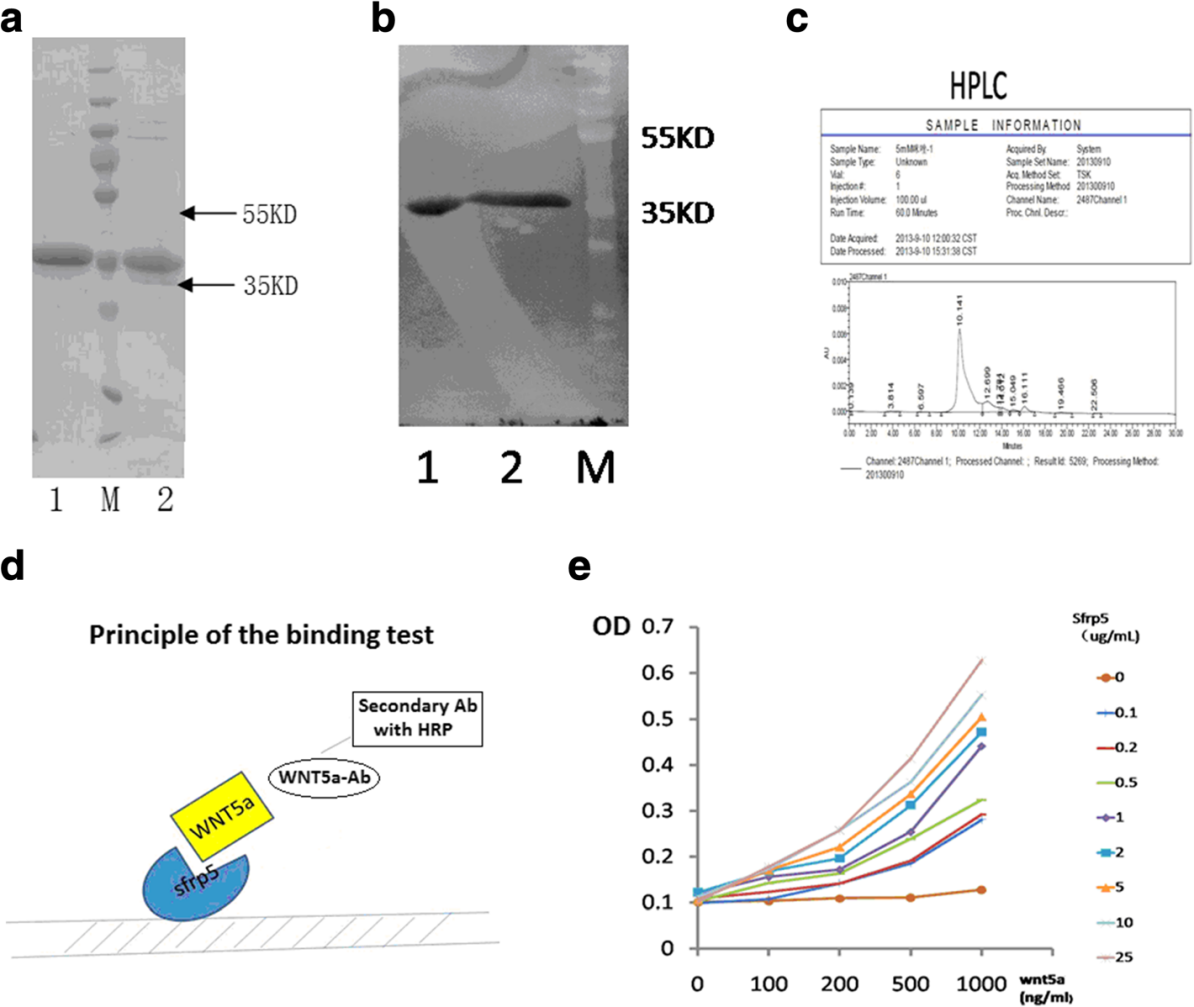
Identification and in vitro activity test of recombinant SFRP5. **a** SDS-PAGE of purified SFRP5 protein. 1. Purified SFRP5 (reductive with DTT), M: Marker (130kd,100kd,70kd,55kd,35kd,25kd,15kd,10kd). 2. Purified SFRP5 (non-reductive) (**b**) western blot of purified SFRP5 protein. 1. Purified SFRP5 (reductive with DTT), 2. Purified SFRP5 (non-reductive), M: Marker (130kd,100kd,70kd,55kd,35kd,25kd,15kd,10kd). **c** HPLC of purified SFRP5 protein. **d** Principle of the binding test of SFRP5 with Wnt-5a. **e** The binding curve shows SFRP5 combines with Wnt-5a specifically in vitro

Recombinant SFRP5 protein was further identified by SDS-PAGE (Fig. 2a), western blot (Fig. 2b), HPLC (Fig. 2c), protein mass spectrometry and Wnt5a-binding test (Fig. 2e). Fig. 2d illustrates the mechanism for verification of the specific binding of recombinant SFRP5 protein to Wnt-5a. When SFRP5 was not coated on the wall of the tube, the absorbance remained at the background level regardless of the concentration of Wnt-5a. When SFRP5 was coated on the wall of the tube, the absorbance increased as Wnt-5a concentration increased. At the same concentration of Wnt-5a, the absorbance increased with the increase of SFRP5 concentration. The recombinant SFRP5 protein is verified to be of high purity and of specific in vitro binding capacity with Wnt-5a. The purity of recombinant SFRP5 protein is 90% identified by HPLC. Its molecule size is 36,096.08 tested by mass spectrometry. In vitro experiments this protein can specifically bind with Wnt5a which verifies its activity in vitro. The endotoxin level of this recombinant protein is 0.01EU/μg-0.1EU/μg and is suitable for animal experiment.

### NASH was induced by MCDD

Mice fed with MCDD developed elevated transaminases and intrahepatic inflammation. The ALT [(315.40 ± 225.80) U/l vs. (40.40 ± 18.70) U/l, *P* < 0.01] and AST [(439.80 ± 151.70) U/l vs. (125.40 ± 18.20) U/l, *P* < 0.01] levels, as well as the intrahepatic steatosis and inflammation scores of MCDD fed mice were significantly higher than controls (Table 1).

**Table 1:** The intrahepatic steatosis and inflammation score after SFRP5 treatment

	Normal diet	MCDD + saline	MCDD + SFRP5
steatosis score	0	2.20 ± 0.63	1.40 ± 0.97*
inflammation score	0	2.00 ± 0.47	1.40 ± 0.70*

*P < 0.05, compared with MCDD + saline group

### Recombinant SFRP5 alleviated the liver injury of NASH

**Fig. 3:**
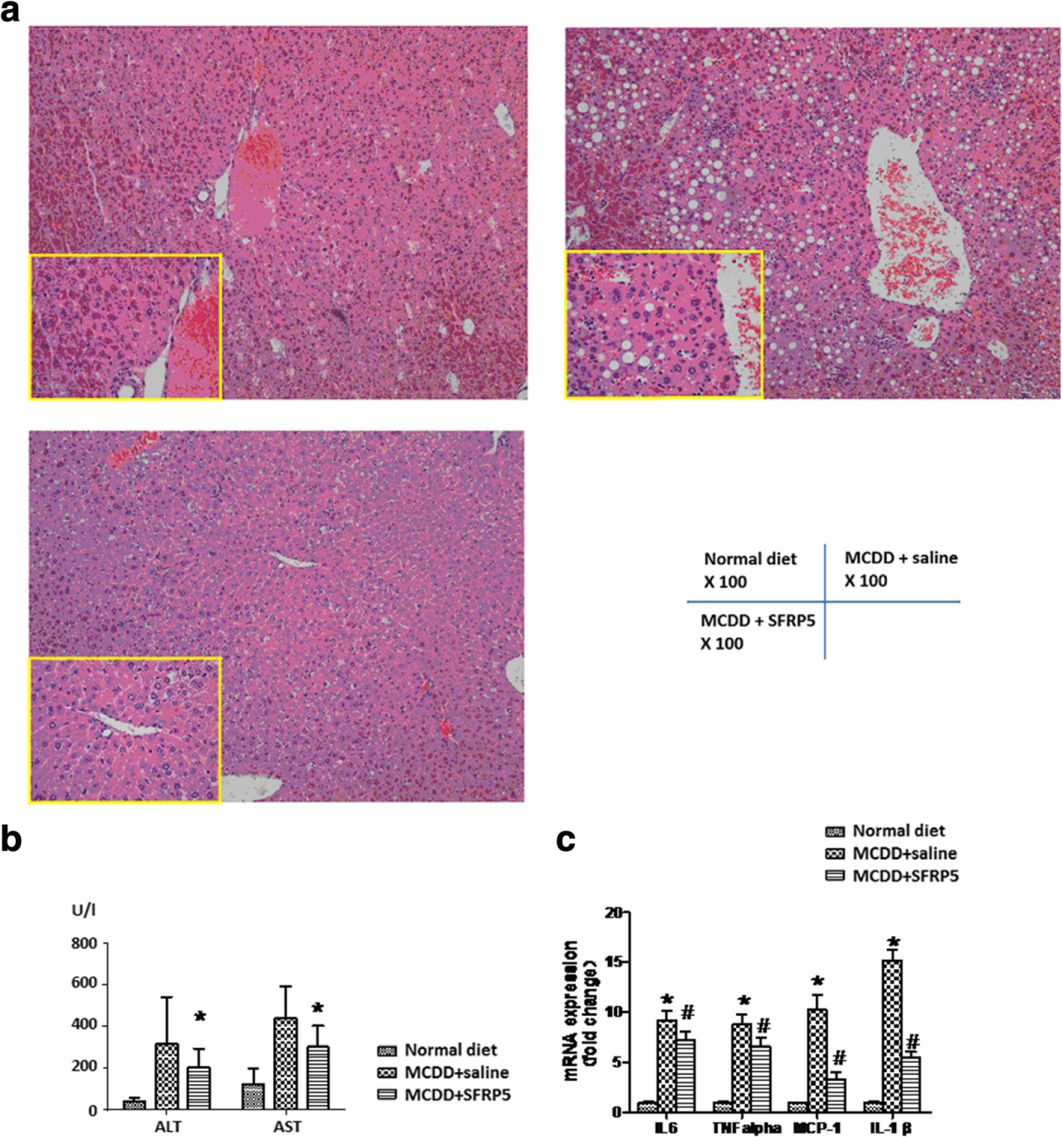
SFRP5 alleviated the intrahepatic steatosis and inflammation of NASH**. a** Under HE staining, the magnification × 100 times. The local magnification of the yellow box is 400 times. **b** SFRP5 alleviated the transaminase elevation induced by MCDD. *P < 0.01, compared with MCDD + saline group. **c** SFRP5 down-regulated the expression of proinflammatory adipokines. *P < 0.01, compared with normal diet group. ^#^ *P* < 0.01, compared with MCDD + saline group

In mice fed with MCDD, the ALT [(202 ± 93.0) U/l vs. (315.40 ± 225.80) U/l, *P* < 0.01] and AST [(302 ± 100.30) U/l vs. (439.80 ± 151.70) U/l, *P* < 0.01] levels of recombinant SFRP5 treatment group were significantly lower than saline control (Fig. 3b). Recombinant SFRP5 intervention decreased liver transaminases and ameliorated MCDD induced liver injury.

### Recombinant SFRP5 alleviated the intrahepatic steatosis and inflammation of NASH

As shown in Fig. 3a and Table 1, mice fed with MCDD developed elevated intrahepatic steatosis (2.20 ± 0.63) and inflammation scores (2.00 ± 0.47), which could be lowered by recombinant SFRP5 treatment (1.40 ± 0.97 and 1.40 ± 0.70, respectively, both *P* < 0.05).

### Recombinant SFRP5 down-regulated the expression of proinflammatory cytokines in liver

After intervention with recombinant SFRP5, the intrahepatic IL-6 (7.21 ± 0.89 vs. 9.23 ± 0.95), TNFα (6.56 ± 0.91 vs. 8.76 ± 1.03), IL-1β (5.48 ± 0.51 vs. 15.10 ± 1.12) and MCP-1 (3.29 ± 0.72 vs. 10.27 ± 1.46) expression were significantly lowered (all *P* < 0.01) on quantitative PCR. Recombinant SFRP5 dramatically down-regulated the expression of proinflammatory cytokines in liver (Fig. 3c).

### Recombinant SFRP5 inhibited Kupffer cell activation

**Fig. 4:**
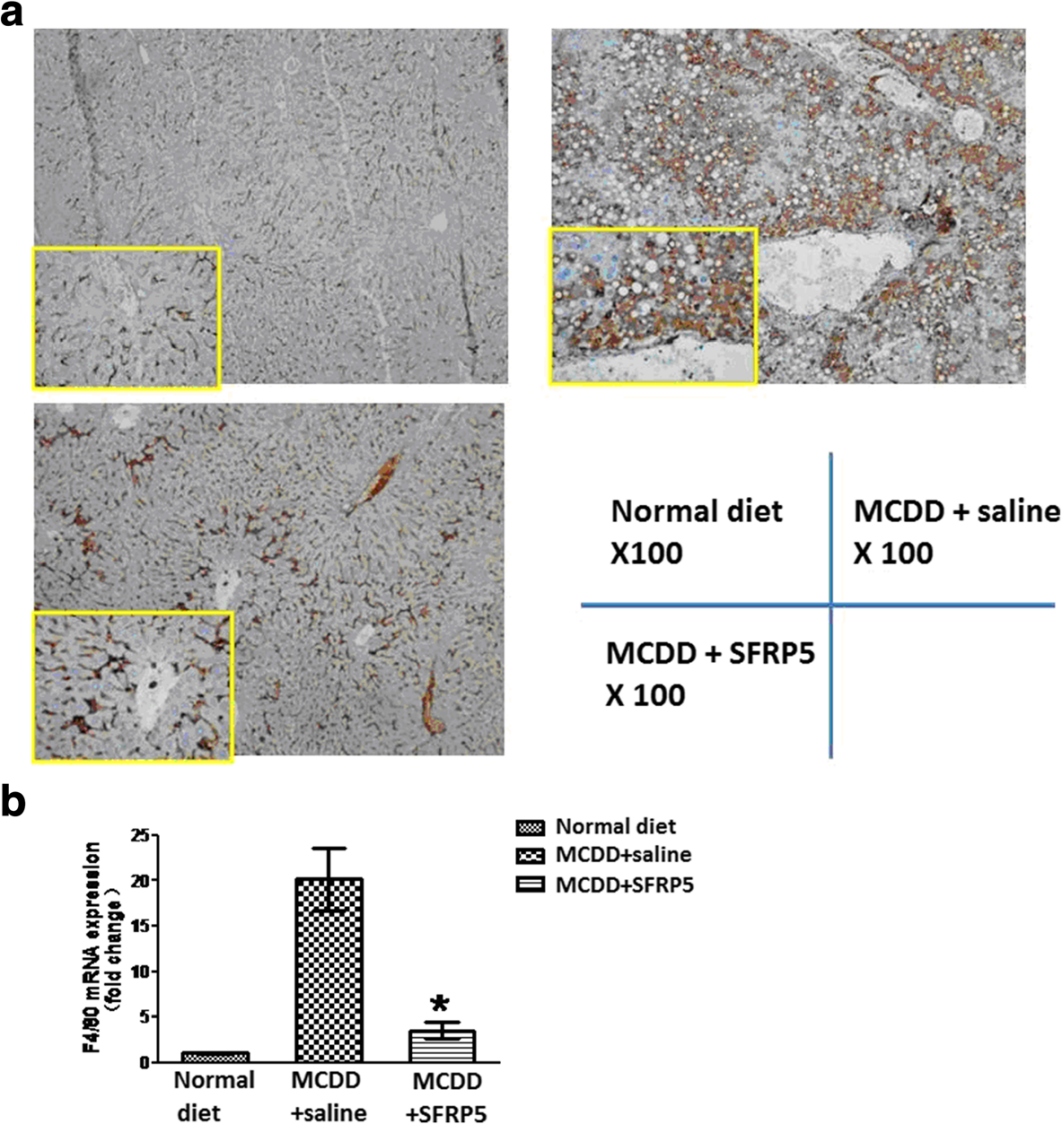
Recombinant SFRP5 inhibited Kupffer cell activation. **a** F4/80 labeled Kupffer cell staining under immunohistochemistry. The magnification is × 100 times, while the local amplification of the yellow box is 400 times. **b** The expression of F4/80 mRNA in liver by quantitative PCR. *P < 0.05, compared with the MCDD + saline group

MCDD fed mice demonstrated Kupffer cell activation. The positive rate of liver F4/80 immunohistochemistry staining in MCDD fed mice was significantly higher than controls. Quantitative PCR showed that the expression of F4/80 mRNA in liver was up-regulated in MCDD fed mice. After SFRP5 treatment, the positive rate of liver F4/80 immunohistochemistry staining and quantitative PCR verified liver F4/80 expression (20.20 ± 3.45 vs. 3.52 ± 0.89, *P* < 0.01) decreased dramatically (Fig. 4a and b), suggesting recombinant SFRP5 treatment could inhibit Kupffer cell activation.

## Discussion

So far, the treatment options for NASH are very limited. The only two effective drugs that have solid clinical evidence are pioglitazone and vitamin E [6, 17]. However, the efficacy of these two drugs on NASH is still unsatisfactory. More in-depth understanding of NASH mechanism and exploration for novel intervention targets is needed. It is not entirely clear about the exact cellular and molecular pathogenesis of NASH, in which multiple pathways are involved. Different stages of NALFD can develop successively or simultaneously, due to environmental factors, genetic heterogeneity, intestinal flora and their complex interactions and dialogue, which could result in simple steatosis, autoimmune system activation, chronic low-grade inflammation, cell death or progressive liver injury [18]. In recently years, the dialogue between adipose tissue and liver has been increasingly valued [19]. Adipose is considered to function not only as an energy storage organ but also as an endocrine organ, in which various adipokines are secreted and involved in the regulation of inflammatory balance and energy homeostasis. For instance, mice with interleukin-6 or tumor necrosis factor α receptor defect are protected from steatosis and hepatitis induced by high fat diet [20]. In contrast, after knockout of the anti-inflammatory adipokine adiponectin, the mice become vulnerable to steatosis, hepatitis and hepatocarcinoma [21–23]. Hence, adipokines may be an important clue for research on the pathogenesis and treatment of NASH, especially those adipokines which are involved in energy regulation and are closely related with intrahepatic inflammation, fibrosis and tumor genesis [24].

SFRP5 is an anti-inflammatory adipokine that regulates metabolic homeostasis. In a previous study [9], *Sfrp5* knockout mice fed high fat diet developed adipose macrophage infiltration, severe glucose intolerance and hepatic steatosis. However, in another study by Mori et al. [25], *Sfrp5* hypomorph mice were resistant to diet-induced obesity and glucose intolerance. *Sfrp5* ^−/−^ mice were generated by replacing the first protein coding exon in the first study, while *Sfrp5* hypomorph mice were generated by chemical mutagenesis leading to a premature stop codon at glutamine 27 in the other study. In addition, the study by Mori et al. showed a compensatory increase in the anti-inflammatory adipokine SFRP1 [26] in *Sfrp5* hypomorph mice, which could explain the different phenotypes in the two studies [9, 25].

Delivery of SFRP5 via adenovirus alleviated glucose intolerance and hepatic steatosis in mice with diet-induced obesity [9]. In spite of the beneficial effect on steatosis, the efficacy of recombinant SFRP5 on intrahepatic inflammation is still unknown. Moreover, the metabolic phenotypes of *Sfrp5* ^−/−^ mice in previous studies were controversial. In light of this, we observed the effect of recombinant SFRP5 protein on MCDD induced NASH in our study.

In the current study, we successfully synthesized recombinant SFRP5 by construction of prokaryotic plasmid expression system. Recombinant SFRP5 protein was further purified and identified by SDS-PAGE, western blot, HPLC, mass spectrometry and Wnt5a-binding test. In order to investigate the effect of recombinant SFRP5 on NASH, we fed mice with MCDD and successfully obtained the animal model of NASH. Intraperitoneal injection of recombinant SFRP5 lowered the serum transaminases, reduced the intrahepatic steatosis and inflammation scores, and inhibited Kupffer cell activation and intrahepatic inflammatory adipokine expression in NASH model mice. High purity of the recombinant SFRP5 protein was obtained. Both in vitro and animal experiments proved its activity.

Our study verifies that recombinant SFRP5 can ameliorate MCDD induced intrahepatic inflammation and steatosis. After intervention with recombinant SFRP5, the intrahepatic inflammation score and intrahepatic IL-6, TNFα, IL-1β and MCP-1 expression were significantly lowered. Moreover, liver F4/80 immunohistochemistry and quantitative PCR illustrated SFRP5 treatment could inhibit Kupffer cell activation. However, the underlying mechanism is to be resolved. Since SFRP5 binds with Wnt-5a, and Wnt-5a promotes the expression of proinflammatory cytokines by macrophages [27], we conclude that SFRP5 alleviates NASH by inhibiting Kupffer cell activation and intrahepatic inflammation. Our research is supported by a study by JJ Fuster et al. [27] where targeted Wnt-5a transgenic and knockout mice were analyzed in a diet induced obesity model. Wnt-5a promotes the expression of proinflammatory cytokines by macrophages in a Jun NH2-terminal kinase-dependent manner, leading to metabolic dysfunction [27].

## Conclusions

Our research suggests that recombinant SFRP5 alleviates MCDD induced intrahepatic inflammation and steatosis. Recombinant SFRP5 could be a novel treatment option for NASH, which needs to be further confirmed by clinical trials.

## Additional file

Additional file 1: Table S1. The intrahepatic steatosis and inflammation scoring system (DOC 35 kb)

## Abbreviations

HPLC: High performance liquid chromatography
MCDD: Methionine choline deficient diet
NAFLD: Non alcoholic fatty liver disease
NASH: Nonalcoholic steatohepatitis
SFRP5: Secreted frizzled-related protein 5
TAL: Tachypleus amebocyte lysate
