# Supplementary material for: Recombinant SFRP5 protein significantly alleviated intrahepatic inflammation of nonalcoholic steatohepatitis
Source: Nutr Metab (Lond). 2017 Aug 15;14:56. doi: 10.1186/s12986-017-0208-0 (PMC5558761; doi:10.1186/s12986-017-0208-0)
Supplement: Additional file 1: Table S1. — The intrahepatic steatosis and inflammation scoring system (DOC 35 kb) [file 12986_2017_208_MOESM1_ESM.doc]

**Additional file 1: Table S1 The** intrahepatic steatosis and inflammation scoring system

|  | Definition | Score |
| --- | --- | --- |
| Steatosis |  |  |
|  | Evaluation of parenchymal involvement by steatosis |  |
|  | <5% | 0 |
|  | 5%-30% | 1 |
|  | 31%-50% | 2 |
|  | 51%-75% | 3 |
|  | >75% | 4 |
| Inﬂammation |  |  |
|  | No inﬂammation | 0 |
|  | Minimal ballooning degeneration of hepatocytes in acinar zone 3, scattered intraacinar foci of spotty necrosis | 1 |
|  | Overt ballooning degeneration of hepatocytes in acinar zone 3, medium amount of intraacinar foci of spotty necrosis, mild to medium portal inﬂammation | 2 |
|  | Extensive ballooning degeneration of hepatocytes in acinar zone 3, extensive intraacinar foci of spotty necrosis, mild to medium portal inﬂammation with/without periportal area inﬂammation | 3 |
